# Supplementary material for: CD8 T Cell Score as a Prognostic Biomarker for Triple Negative Breast Cancer
Source: Int J Mol Sci. 2020 Sep 22;21(18):6968. doi: 10.3390/ijms21186968 (PMC7555570; doi:10.3390/ijms21186968)
Supplement: Supplementary file 1 [file ijms-21-06968-s001.pdf]

SUPPLEMENTAL MATERIALS

Oshi et al. "CD8 T cell score as a prognostic biomarker for triple negative breast cancer"

Contents

Table S1: Symbols and names of genes that constitute the CD8 T cells score.
Figure S1: Association of CD8 score with disease-specific survival in high and low *MKI67* expression of ER-positive breast cancer, and luminal A and B subtypes.

Table S1. Symbols and names of genes that constitute the CD8 T cells score.

Gene Symbol	Gene Name
AAK1	AP2 associated kinase 1
APBB1	amyloid beta precursor protein binding family B member 1
ARHGEF1	Rho guanine nucleotide exchange factor 1
BTN2A1	butyrophilin, subfamily 2, member A1
C7orf26	chromosome 7 open reading frame 26
CA6	carbonic anhydrase 6
CASP8	caspase 8
CBY1	chibby family member 1, beta catenin antagonist
CCDC25	coiled-coil domain containing 25
CCDC53	coiled-coil domain containing 53
CCR7	C-C motif chemokine receptor 7
CD160	CD160 molecule
CD27	CD27 molecule
CD3D	CD3d molecule
CD7	CD7 molecule
CD8A	CD8a molecule
CD8B	CD8b molecule
CD96	CD96 molecule
CEPT1	choline/ethanolamine phosphotransferase 1
CIAPIN1	cytokine induced apoptosis inhibitor 1
CLUAP1	clusterin associated protein 1
COG2	component of oligomeric golgi complex 2
COPZ1	coatomer protein complex subunit zeta 1
CRTAM	cytotoxic and regulatory T cell molecule
CTSW	cathepsin W
CX3CR1	C-X3-C motif chemokine receptor 1
DHX15	DEAH-box helicase 15
DIDO1	death inducer-obliterator 1
DNAJB1	DnaJ heat shock protein family (Hsp40) member B1
DPP8	dipeptidyl peptidase 8
DSC1	desmocollin 1
EEF1D	eukaryotic translation elongation factor 1 delta
EML3	EMAP like 3
FAM134C	family with sequence similarity 134, member C
FBXW4	F-box and WD repeat domain containing 4
FKTN	fukutin
FNBP4	formin binding protein 4
FTO	FTO alpha-ketoglutarate dependent dioxygenase

GGNBP2	gametogenetin binding protein 2
GIMAP4	GTPase, IMAP family member 4
GJC2	gap junction protein gamma 2
GZMH	granzyme H
GZMK	granzyme K
GZMM	granzyme M
HNRNPA0	heterogeneous nuclear ribonucleoprotein A0
HNRNPL	heterogeneous nuclear ribonucleoprotein L
IL16	interleukin 16
IPCEF1	interaction protein for cytohesin exchange factors 1
IRF3	interferon regulatory factor 3
KLHL3	kelch like family member 3
KLRB1	killer cell lectin like receptor B1
KLRG1	killer cell lectin like receptor G1
KRT2	keratin 2
LAIR2	leukocyte-associated immunoglobulin-like receptor 2
LSM14A	LSM14A mRNA processing body assembly factor
LY9	lymphocyte antigen 9
MED17	mediator complex subunit 17
MKRN2	makorin ring finger protein 2
MMP19	matrix metallopeptidase 19
MSL3	male-specific lethal 3 homolog
MTRF1	mitochondrial translation release factor 1
MYOM1	myomesin 1
NAA16	N(alpha)-acetyltransferase 16, NatA auxiliary subunit
NDFIP1	Nedd4 family interacting protein 1
NDUFS2	NADH:ubiquinone oxidoreductase core subunit S2
NFKB1	nuclear factor kappa B subunit 1
NKRF	NFKB repressing factor
NPAT	nuclear protein, coactivator of histone transcription
NPRL2	NPR2 like, GATOR1 complex subunit
PCNT	pericentrin
PFN2	profilin 2
PLCG1	phospholipase C gamma 1
PLXDC1	plexin domain containing 1
POLR3E	RNA polymerase III subunit E
POP5	POP5 homolog, ribonuclease P/MRP subunit
PRL	prolactin
PRMT2	protein arginine methyltransferase 2
PRPF4B	pre-mRNA processing factor 4B
PSD	pleckstrin and Sec7 domain containing
PTGDR	prostaglandin D2 receptor
PTPN4	protein tyrosine phosphatase non-receptor type 4
PURA	purine rich element binding protein A
RAPGEF6	Rap guanine nucleotide exchange factor 6
RASA2	RAS p21 protein activator 2
RBL2	RB transcriptional corepressor like 2
RBM34	RNA binding motif protein 34
RING1	ring finger protein 1
RNF113A	ring finger protein 113A
RPL37A	ribosomal protein L37a
RWDD3	RWD domain containing 3
S100B	S100 calcium binding protein B
SDAD1	SDA1 domain containing 1
SDCCAG3	serologically defined colon cancer antigen 3
SFPQ	splicing factor proline and glutamine rich

SHANK1	SH3 and multiple ankyrin repeat domains 1
SIRPG	signal-regulatory protein gamma
SLC1A7	solute carrier family 1 member 7
SSTR3	somatostatin receptor 3
TBCC	tubulin folding cofactor C
TMEM41B	transmembrane protein 41B
TOMM7	translocase of outer mitochondrial membrane 7
TRAF3IP3	TRAF3 interacting protein 3
TSPAN32	tetraspanin 32
TTN	titin
UBE2Q1	ubiquitin conjugating enzyme E2 Q1
UBQLN2	ubiquilin 2
USP47	ubiquitin specific peptidase 47
UTP20	UTP20 small subunit processome component
WDR82	WD repeat domain 82
YLPM1	YLP motif containing 1
ZBTB11	zinc finger and BTB domain containing 11
ZC3HAV1	zinc finger CCCH-type containing, antiviral 1
ZNF154	zinc finger protein 154
ZNF200	zinc finger protein 200
ZNF611	zinc finger protein 611
ZNF639	zinc finger protein 639

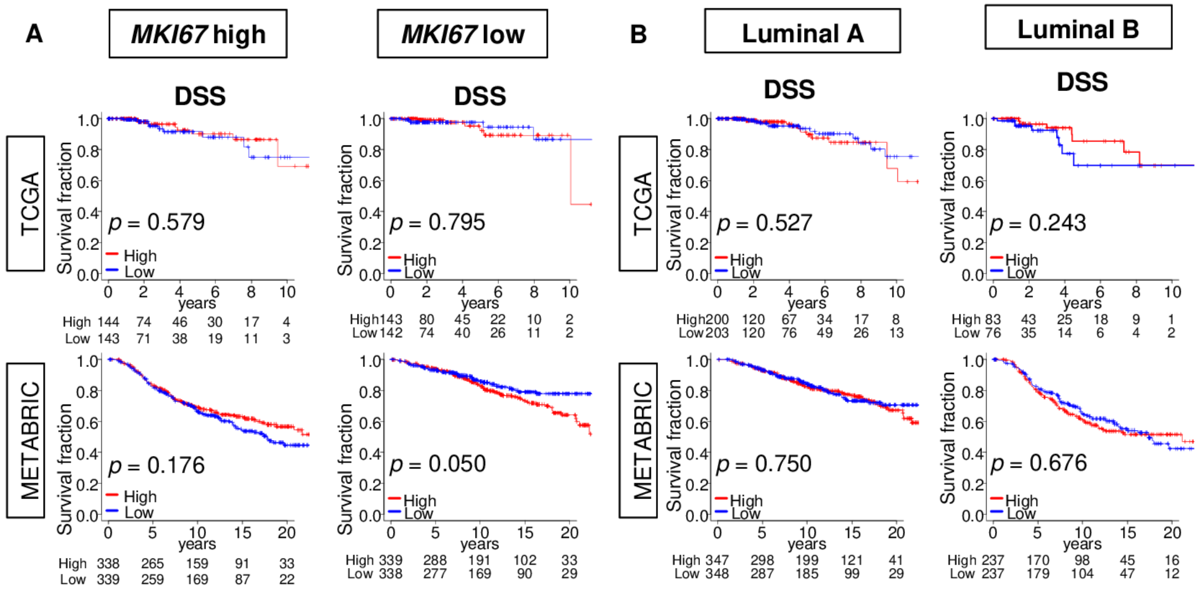

Figure S1. Association of CD8 score with disease-specific survival in high and low *MKI67* expression of ER-positive breast cancer, and luminal A and B subtypes**. (A)** Disease-Specific Survival (DSS) of CD8 score low (blue) vs. high (red) within *MKI67* high and low in ER-positive/HER2-pegative breast cancer in both TCGA and METABRIC**. (B)** Disease-Specific Survival (DSS) of CD8 score low (blue) vs. high (red) within luminal A and luminal B in both cohorts. Kaplan-Meier survival curves with log-rank test was used to the analysis.
